# McGill University Proceedings for the Year 1894–95

**Published:** 1896-01

**Authors:** 


### PROCEEDINGS FOR THE YEAR 1894-95 OF THE SOCIETY FOR THE STUDY OF COMPARATIVE PSYCHOLOGY IN CONNECTION WITH THE FACULTY OF COMPARATIVE MEDICINE AND VETERINARY SCIENCE OF THE McGILL UNIVERSITY, MONTREAL.

To economize space it may be stated that the Society's meetings were held at intervals of about two weeks from October to March, inclusive, in the lecture-room of the Faculty, and that the President, Prof. Wesley Mills, M.D., D.V.S., etc., presided at each meeting.

The Society met for the purpose of electing officers for the year 1895, the President, Dr. Wesley Mills, occupying the chair. The following were elected: Honorary President, Dr. Duncan McEachran; President, Dr. Wesley Mills; First Vice-President, Dr. M. A. Dawes; Second Vice-President, Mr. Sherman Cleaves; Secretary-Treasurer, Mr. C. A. Boutelle; Corresponding Secretary, Mr. A. Cowan; Press Reporter, Mr. Harri Dell. Twenty new members were nominated for election at the next meeting, at which Dr. Dawes will deliver the inaugural address. The Honorary President, Dr. McEachran, and the President, Dr. Wesley Mills, addressed the meeting, stating the objects of the Society, pointing out the field for research and the necessity for individual observation. Mr. C. H. Zink read a paper published in *Forest and Stream*, describing the extent to which a certain retriever was able to understand human language. The writer based his conclusions upon original and careful investigation of the psychic phenomena manifested by his canine companion. A list of words was given, the meaning of which the animal appeared to thoroughly understand. The paper was well written, the subject handled in an unprejudiced manner, and proved of exceeding interest to those present.

The Society, which is in a flourishing condition, consists principally of professors and students of the Faculty of Comparative Medicine, having for its object the study of the psychic phenomena of the lower animals. The Society possesses a small, but valuable, and increasing library pertaining to psychology and kindred subjects.

After routine general business, the Society proceeded to the election of new members, twenty being added to the roll.

Concerning the prizes offered, it was agreed that all those in competiton for the same should be in the hands of the Secretary for transmittal to the committee on or before March 1, 1895.

Dr. M. A. Dawes then delivered the inaugural address, in which he pointed out the practical applications of a knowledge of psychic phenomena. In investigating these phenomena, we would, at the same time, be increasing our powers of observation in health and disease. Thus a knowledge of comparative psychology would aid us in clinical examinations, and, furthermore, lead us to a more kindly treatment of our animal friends.

Mr. C. H. Zink then read a paper, published in the *American Naturalist*, on the "Habit of Amusement in Lower Animals," in which were described the peculiar postures and behavior of animals low in the biological scale. The writer had seen tiny *rhizopoda* chasing each other around as if in a game of "tag," also *rotifera* dancing by themselves. He had also witnessed the meeting of two snails, who performed a series of peculiar motions, bowing their heads and waving them from side to side. The movements described were thought to be in no way connected with mating, because having examined swarms of *diptera* dancing in the sunlight, also forms of *coleoptera* engaged in playful gambols, the author found them in each case to be females. He also described the apparently malicious sport of a certain *pulex irritans* with a sleeping comrade, and expressed himself as convinced that "every animal at some period in life has a true appreciation of psychical amusement."

After the reading of the paper the President stated that he had himself witnessed actions among the *rotifera* and other minute forms of animal life which indicated what, in higher forms, we would call exuberance of animal spirits.

Mr. Inglis then read a paper, published in the *American Stockkeeper* and copied by that journal from the *British Fancier*, on the subject of "Dogs and Music." The physiological effect of music was described. In the opinion of the writer, dogs first fear, then become interested, and finally acquire a liking for music, preferring a reed instrument to a stringed one. They are exceedingly sensitive to discord, one case in particular being cited of a dog in Darmstadt which, while appearing to enjoy music, gave vent to dismal howls whenever a discordant note was sounded. An experiment at the London Zoölogical Gardens, with a violin, was described, showing the difference in the development of the musical sense in different animals. Wolves were terrified, as were also the jackals, but in a less degree, while the foxes and dingoes appeared to experience at first painful followed by pleasurable sensations. The attachment of shepherd dogs to the strains of the bagpipes was also noted, and the question raised of the influence of music on sick dogs.

In the discussion which followed, Mr. Zink criticised the opinions of the writer, directing attention to the difficulty, owing to obvious conditions and circumstances, of arriving at positive conclusions regarding the effect of music on the lower animals. The enlivening effect of music upon melancholy had been experienced probably by most of those present.

Dr. Baker suggested that experiments relating to the effect of music on the lower animals in disease be carried out in the college hospital.

While the lower animals, as a rule, do not produce music, it is evident that in many cases they appreciate it. The subject of street music arose out of the discussion, and it was thought by the President that while, as a rule, it was not of a very high order, still it is not without its beneficial psychic effect on a class of people who are unable to hear better.

After further remarks by the President, the Society adjourned.

Subsequent to the reading of the minutes of the previous meeting, roll-call, and the transaction of general business, the members listened to a paper on the subject of "Fear," by Mr. C. H. Zink. After referring briefly to the physical phenomena of fear, as witnessed in the exhibition of palpitation of the heart, the inhibition of respiration, and the changes in the secretions, etc., the writer dealt with the psychical aspect of the subject. The manifestations of fear in the higher forms of animal life would naturally be most complex, and relatively decrease in complexity in the lower forms. In the amœba, the nearest approach to fear lies in its irritability, such as is displayed when pricked by a needle. While the term fear cannot be applied to this irritability, it is probably a forerunner of that emotion. This protoplasmic irritability was compared to the apex of an inverted pyramid, of which fear was one of the stones resting thereon. With extension and differentiation not only can fear be accounted for, but many other physical phenomena. Animals low in the biological scale receive impressions by direct contact or chemiotactic influences; then, progressing up the scale, we find developing special senses, each succeeding one being more complex. In insects, sight is an important factor in producing the element of fear; in fishes, a noise or light will cause them to scatter in all directions with apparent alarm; in birds with higher development, fear becomes more complex and easily recognized, while in mammals this complexity is greatly increased. One can now differentiate between curiosity, alarm, fear, and terror, even to loss of consciousness and death, these terms being, in the opinion of the writer, merely degrees or gradations of fear. The value of fear to animals, the cause and conditions under which it was manifested, were next considered. Throughout the entire animal kingdom, fear prompts the organism to escape from threatened danger, whether this escape be effected by locomotion, feigned death, or any other strategic mental measures. The causes of fear were summed up to be "a knowledge of danger, derived by the animal from the means at its command, and depending, therefore, on the complexity of its development." The writer had witnessed the feigning due to fear on the part of a very young calf, and contrasted this fact with the anecdotes, as told by Arctic explorers, of the curiosity and lack of fear as displayed by seals. With increased complexity of the nervous system is a corresponding complexity of conditions, and in addition to the psychical phenomena previously mentioned, we find changes in the circulatory, motor, and sensory organs, paralysis, loss of consciousness, and even death. It was thought that the unknown, rather than that of which the animal is cognizant, is most productive of fear. In some animals curiosity is excited; this leads to investigation, and this, while adding in a sense to the knowledge in general, leads to a realization of much that is unknown, and the animal fears. Fear, then, is the shock to the directing part of the organism resulting from environmental influences to which the animal is unaccustomed. "When the first molecule of matter became imbued with the breath of life there was something—shall we call it environmental?—which, causing variation, joined with natural selection and survival of the fittest, made improvement possible, gave an opportunity to grow, and in this provision we see the origin of fear and all other emotions; in fact, all mental phenomena."

An animated discussion followed the reading of the paper. The President referred in eulogistic terms to the original and scientific treatment of the subject by Mr. Zink. Mr. Thurston asked how the writer would explain the feigning of the calf which had been referred to in the paper. This fear and feigning were thought to be due to instinct. The President, however, thought that no instinct is perfect without experience, and that instinct in the mind corresponds somewhat to reflexes in the body. Cases of hereditary instinctive fear of bears in horses were related by Messrs. Hart and Parker. Mr. McKeracher reported an instance of a mare becoming restive from no apparent cause until a bear was discovered in the vicinity. Mr. Hilliard had known of cattle greatly excited and unwilling to proceed along a road which had been crossed by a bear half an hour previously. In these two cases fear was evidently produced through the sense of smell. Mr. Ness related an interesting case of some horses confined in a field showing great fear at the sight of a bear being led along the road, but, strange to say, the young colts appeared to exhibit only curiosity. Mr. Dell had seen terror produced in a horse at the sight of a black sheepskin, although the animal was not afraid of the skins of other animals. This was supposed to be due to the law of association, the animal having, probably, at some previous time, been frightened by something of a similar nature.

After further remarks by the President, the Society adjourned to meet again in two weeks.

After roll-call and the reading of the minutes of the previous meeting, the President announced the receipt of several new works bearing on psychology and anthropology, which were added to the library.

The Society was also presented by Dr. Mills with copies of some of his own works, including a paper read before the Royal Society on "Squirrels, their Habits, and Intelligence, with special reference to Feigning."

Mr. E. C. Thurston read a paper on "Instinct and Intelligence of Animals," in which he clearly defined each of these terms, claiming that a definite line of demarcation might be drawn between them. Many actions of the lower animals have been ascribed to instinct, but on careful investigation one is obliged to admit, however unwillingly, that they possess a mind capable of limited degree of reflection and comparison, and are able to draw conclusions from facts considered. The great barrier to a more complete understanding of the psychic nature of the lower animals lies in the fact that they lack, almost entirely, the power of speech. The same tests, however, which prove the intelligence of the child before it has learned to speak, will demonstrate, when applied to the lower animals, the existence in them of a similar power of comprehending the meaning of words. The intelligent actions of the terrier "Jack," an attache of the college, were next referred to in detail, it being clearly demonstrated that he was able to communicate his wishes to those around him.

Mr. E. H. Lehnert followed with a paper on "The Relation of Reflex Action to Instinct." A reflex act in its simplest form is a single act, *e.g.*, contraction following upon a single irritation. This simple form of reflex, giving rise to the lowest form of psychical activity, would therefore approach nearest to machine-like action. The reflex act proper occurs only in those animals possessed of a nervous system, those which are not mere masses of irritable and contractile protoplasm. While the simpler reflexes may be physical processes, the higher ones involve a complex series or combination of physical processed and have psychical equivalents, physical acts being associated with complex psychical processes, the dividing line between physical and psychical life cannot always be clearly defined, and must in a measure be arbitrary. The life-processes in the highly organized animal were shown to be regulated reflexes, and might be far removed from instincts which were "the result of accumulated experiences along certain lines." The manifestations of instinct in the fly-catcher in the nest were referred to in support of this statement. There can be no doubt that the more frequently psychical states occur in a certain order the more inseparable they become, and also that this tendency, even in a slight degree, may be hereditary, and we will see that instinct is the product of accumulated experiences; for if the experiences of the species remain the same, each successive generation will transmit to offspring a somewhat increased tendency, and this tendency will ultimately result in an automatic relation between the nervous action and external influences perpetually experienced. If then some change takes place in the environment so that new external relations are brought to bear on the animal, and the organism be susceptible to these, a new series of psychical states will arise, at first being a reflex, and later by generations of repeated experiences the tendency becomes organic or automatic, and hence an instinct.

A short discussion of the papers followed. Mr. Zink felt that the popular idea of instinct had received a severe blow by the arguments brought forward in Mr. Thurston's paper. The President explained the uselessness of attempting to train dogs for the field unless they possessed at least an element of hunting instinct. He referred, however, to the case of a St. Bernard and a setter which had learned by imitation to catch vermin, but in all dogs there is the original tendency to hunt, though in some latent. An inquiry from Mr. Baldwin as to the extent to which a dog might really understand the meaning of words evoked an animated discussion, in which several of the members took part.

At the request of the President, Mr. Thurston read an article from the *Dog Fancier* on "Animal Psychology (a protest)," in which the writer expressed his peculiar convictions on the subject. This writer is agnostic as to dogs' reasoning, etc.

Mr. Zink read a recent article from the pen of Mr. George Mivart on the "Distinction Between the Human and Animal Mind," but owing to the lateness of the hour the discussion of these papers was deferred until the next meeting.

A letter was read from Mr. E. K. Whitehead, of Denver, Col., confirming the statements in the "Tale of a Dog," which appeared recently in *Forest and Stream*. The photograph of this remarkable animal was exhibited to the members, after which the meeting adjourned.

(To be concluded.)

